# Hygroscopic Behaviour and Diffusion Characteristics of Flexible TPU Materials Fabricated by FDM for Potential Biomedical Applications

**DOI:** 10.3390/polym18111392

**Published:** 2026-06-04

**Authors:** Nikola Šimunić, Tihana Kostadin, Josip Hoster, Dino Obranović

**Affiliations:** Mechanical Engineering Department, Karlovac University of Applied Sciences, Trg J.J. Strossmayera 9, 47000 Karlovac, Croatia

**Keywords:** additive manufacturing, fused deposition modeling (FDM), thermoplastic polyurethane (TPU), hygroscopic behaviour, diffusion coefficient, water absorption, swelling, saline immersion

## Abstract

Flexible thermoplastic polyurethane (TPU) materials fabricated using fused deposition modeling (FDM) are increasingly used in engineering and biomedical applications where exposure to moisture is unavoidable. However, the relationship between material hardness, water absorption, diffusion behaviour, and dimensional stability remains insufficiently understood and investigated. In this study, the hygroscopic behaviour of eight commercially available TPU filaments (60A–98A Shore hardness) was systematically investigated. Specimens were produced using an FDM 3D printer under controlled processing conditions and immersed in physiological solution (0.9% NaCl) for up to 96 h. Water absorption, dimensional changes, and diffusion characteristics were analyzed. Diffusion coefficients were determined using the Fickian diffusion model based on the initial stage of water uptake. The results suggest a transition in behaviour between lower- and higher-hardness materials. Softer TPU materials (60A–85A) exhibited higher water absorption (up to ~1.80%) and an apparent linear trend between hardness and absorption within the investigated material group (R^2^ = 0.999). In contrast, higher-hardness materials (89A–98A) showed lower absorption (~1.18–1.42%) and a weaker apparent relationship with hardness (R^2^ = 0.4214). Diffusion coefficients ranged from 1.40 × 10^−13^ to 3.40 × 10^−12^ m^2^ s^−1^, with no monotonic dependence on hardness. Additionally, no clear correlation between diffusion kinetics and equilibrium absorption or volumetric expansion was observed. These findings indicate that hygroscopic behaviour of FDM-printed TPU materials cannot be reliably predicted based solely on hardness, and that diffusion, absorption, and swelling may be influenced by different mechanisms. The identified transition from hardness-dependent to behaviour potentially influenced by material structure provides new insight for the design and selection of flexible polymer components in moisture-exposed environments, particularly in biomedical applications.

## 1. Introduction

Additive manufacturing (AM) technologies have significantly transformed modern manufacturing by enabling the production of complex geometries directly from digital models. Among the various AM processes, fused deposition modeling (FDM) has become one of the most widely used techniques due to its accessibility, relatively low cost, and compatibility with a broad range of thermoplastic materials [1,2,3]. In the FDM process, a thermoplastic filament is melted and extruded through a heated nozzle, depositing material layer-by-layer to form a three-dimensional object. This manufacturing approach allows rapid prototyping and production of customized components with complex shapes that would be difficult to achieve using conventional manufacturing methods [1,2].

In recent years, flexible polymer materials have attracted considerable attention in FDM applications. Materials such as thermoplastic polyurethane (TPU), thermoplastic elastomers (TPE), and styrene–ethylene–butylene–styrene (SEBS) exhibit high elasticity, good abrasion resistance, and favourable fatigue properties [4,5]. These characteristics make them particularly suitable for applications requiring flexible or deformable components. Consequently, flexible polymers produced using FDM technology are increasingly used in a variety of fields, including soft robotics, wearable electronics, flexible sensors, and biomedical engineering [6,7].

The biomedical sector represents one of the fastest growing areas for AM technologies. FDM-printed polymers are commonly used for the fabrication of anatomical models, surgical planning tools, orthotic devices, and customized medical aids [6,8]. Flexible polymer materials are especially interesting in biomedical applications because their mechanical properties may partially mimic those of soft biological tissues. For example, flexible polymers have been investigated for the fabrication of dental models, soft tissue simulators, and various patient-specific medical devices [6].

However, materials intended for biomedical environments are frequently exposed to moisture or biological fluids. Water absorption may significantly influence the physical and mechanical properties of polymer materials. Hygroscopic behaviour can lead to swelling, dimensional instability, and potential degradation of mechanical properties [9,10,11]. These effects are particularly important for components that must maintain dimensional accuracy and stability during prolonged exposure to humid environments or fluids. In biomedical environments, materials are commonly exposed to physiological fluids rather than pure water; therefore, the use of saline solution enables a more realistic assessment of hygroscopic behaviour under application-relevant conditions.

The hygroscopic behaviour of polymers is influenced by several factors, including their chemical structure, polarity of polymer chains, and degree of crystallinity. Materials containing polar functional groups tend to interact more strongly with water molecules, which can increase moisture absorption [9]. TPU’s, for example, contain polar urethane groups that may promote water uptake through hydrogen bonding mechanisms [10]. In contrast, polymers composed predominantly of non-polar hydrocarbon chains, such as SEBS, generally exhibit lower affinity for water and therefore reduced hygroscopicity [5].

In addition to intrinsic material properties, the manufacturing process itself can also influence water absorption behaviour. Components produced using FDM technology possess a layered structure composed of extruded filaments. Imperfect bonding between adjacent layers and the presence of microscopic voids or inter-filament gaps may facilitate fluid penetration into the internal structure of the material [11,12]. As a result, FDM-printed parts may exhibit different hygroscopic behaviour compared with conventionally manufactured polymer components.

Although the mechanical behaviour of FDM-printed polymers has been widely investigated, comparatively fewer studies have examined the hygroscopic properties of flexible polymer materials fabricated using this technology. Understanding the interaction between flexible polymers and moisture is important for evaluating long-term stability and performance in moisture-exposed and biomedical-related environments [8,12].

Despite increasing interest in FDM-printed flexible polymers, the relationship between Shore hardness, diffusion kinetics, water absorption, and dimensional stability remains insufficiently understood. This is particularly evident for materials exposed to humid or physiological-like conditions.

It should also be noted that commercially available TPU materials may differ not only in Shore hardness, but also in chemical formulation, including soft/hard segment ratio, polyol chemistry, additive content, and microstructural characteristics associated with different manufacturers and processing conditions. Consequently, hardness in the present study should not be interpreted as a strictly independent variable, but rather as an indicative comparative parameter representing a broader set of material characteristics. Therefore, the objective of this work is primarily comparative and phenomenological, with emphasis placed on identifying general trends in hygroscopic behaviour across a wide range of commercially available TPU materials.

Therefore, the aim of this study is to systematically investigate the hygroscopic behaviour of FDM-printed TPU materials across a wide hardness range. Particular emphasis is placed on analysing the relationship between water absorption, diffusion characteristics, and dimensional stability. Special attention is given to identifying potential transitions in governing mechanisms across different hardness ranges, thereby contributing to a broader comparative understanding of hygroscopic behaviour in flexible FDM-printed polymers.

## 2. Materials and Methods

### 2.1. Materials

Eight commercially available flexible polymer filaments were selected for the experimental investigation (Table 1). The materials consisted primarily of TPU filaments with Shore hardness values ranging from 60A to 98A, sourced from multiple manufacturers. This selection enabled the analysis of mechanical and hygroscopic behavior across a broad range of material stiffness relevant to FDM applications. The investigated materials originated from different manufacturers and may therefore differ in composition and processing characteristics. The materials were chosen due to their widespread use in AM and their potential suitability for flexible biomedical components. All materials were supplied as 1.75 mm diameter filaments and processed following the manufacturers recommendations.

### 2.2. Specimen Preparation

Test specimens were fabricated using FDM technology. A desktop FDM 3D printer (Original Prusa i3 MK3S+, Prusa Research a.s., Prague, Czech Republic) was used to produce square specimens with dimensions of 40 mm × 40 mm × 2 mm (*x* × *y* × *s*). All specimens were manufactured under comparable processing parameters, following the manufacturers’ recommendations, to ensure consistency between materials (Table 2). All specimens were printed in a flat orientation using a rectilinear raster pattern with fully solid top and bottom layers under ambient laboratory conditions (22 ± 1 °C). Retraction and cooling settings were adjusted according to manufacturer recommendations to ensure stable extrusion and minimize printing artefacts in flexible TPU materials. Nevertheless, slight differences in optimal processing conditions between individual materials could not be completely eliminated, since each commercially available filament possesses specific manufacturer-recommended printing parameters. Detailed compositional information such as hard-segment content, plasticizer concentration, and exact TPU chemistry was not consistently available from manufacturer datasheets for all investigated materials. For each material, five specimens (*n* = 5) were produced and subsequently tested.

After printing, the specimens were conditioned and dried in a laboratory oven (Memmert GmbH, Co. KG, Schwabach, Germany; U/UF series) at 55 °C for 24 h to remove residual moisture. The mass of each specimen was then recorded as the initial dry mass (mo). Mass measurements were performed using an analytical balance (KERN & SOHN GmbH, Balingen, Germany) with a readability of ±0.0001 g. All measurements were performed at ambient laboratory conditions (22 ± 1 °C, ambient humidity).

### 2.3. Hardness Measurements

Shore hardness of the investigated materials (TPU) materials was experimentally determined using a Zorn Stendal DDR durometer (Zorn Stendal DDR, Stendal, Germany) in accordance with ISO 868 [13]. The measurements were performed prior to immersion in physiological solution. Due to the elastic nature of the materials, a fixed dwell time of 3 s was applied before reading the hardness value, in accordance with ISO 868. To improve measurement reliability, five measurements were performed at different locations on each specimen, and the average value was reported.

Five specimens were tested for each material, and five hardness measurements were performed on each specimen at different locations, resulting in a total of *n* = 25 measurements per material. The repeated measurements performed on the same specimen were used primarily to assess local variability and measurement repeatability and should therefore not be interpreted as fully independent statistical replicates.

### 2.4. Hygroscopicity Testing

**Figure 1:**
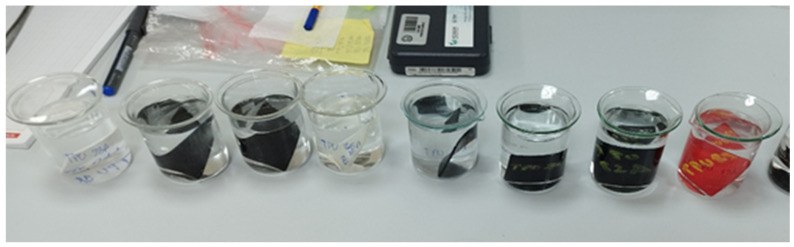
A batch of specimens was placed in glass containers filled with physiological solution.

Hygroscopic behaviour was evaluated by immersing the specimens in physiological saline solution (0.9% NaCl) at room temperature. The solution was selected to simulate a simplified biological environment (Figure 1). The testing procedure was conducted in accordance with ISO 62:2008 [14] (Plastics–Determination of water absorption) for the evaluation of moisture uptake in polymer materials.

The selected immersion intervals (1, 6, 24, and 96 h) were chosen to capture both the initial stage of moisture uptake and the subsequent evolution toward longer-term absorption behaviour. Therefore, the applied methodology should be interpreted as a comparative adaptation of the standard intended for diffusion and hygroscopicity assessment of FDM-printed TPU materials rather than as a full equilibrium water absorption protocol according to ISO 62.

Specimens were immersed for predefined time intervals of: 1 h, 6 h, 24 h and 96 h. After each interval, specimens were removed from the solution, gently dried using paper towels to remove surface liquid, and weighed using an analytical balance.

The percentage of absorbed moisture was calculated using (1):

(1) $$x_{i}=\frac{m_{t,i}-m_{o}}{m_{o}}\times 100\%$$

where

*x_i_*—moisture absorption [%],
*m*_t,i_—specimen mass after immersion time [g],
*m*_o_—dry mass [g].

### 2.5. Dimensional Measurements

In addition to mass measurements, specimen dimensions were recorded in order to monitor potential swelling effects. Length (*x*), width (*y*), and thickness (*s*) were measured using a digital caliper (Mitutoyo, Japan; accuracy ±0.01 mm). Surface area and volume were calculated using standard geometric relations.

The surface area and volume of the specimens were calculated using Equations (2) and (3) to monitor the dimensional stability of the samples.

Surface area equation:

*A* = *x* × *y* (2)

where:

*A*—specimen surface area [mm^2^],
*x*—specimen width [mm],
*y*—specimen length [mm].

Volume equation:

*V* = *A* × *s* (3)

where:

*V*—specimen volume [mm^3^],
*s*—specimen thickness [mm].

### 2.6. Diffusion Modeling

The diffusion coefficient of water in the investigated TPU materials was estimated based on the initial stage of water absorption, assuming Fickian diffusion behaviour [15]. For a plane sheet geometry, the early-time solution of Fick’s second law can be expressed as (4):

(4) $$\frac{M_{t}}{M_{\infty }}=\frac{4}{L}\sqrt{\frac{D\cdot t}{\pi }}$$

where:

*M_t_*—mass of absorbed substance (e.g., water) at time *t* [g],
*M*_∞_—mass at saturation (equilibrium value) [g],
*D*—diffusion coefficient, [m^2^ s^−1^],
*t*—time, [s]
*L*—specimen half-thickness [m].

In this study, specimens with a thickness of 2 mm were used, resulting in *L* = 0.001 m.

The diffusion coefficient D was determined from the slope of the linear region of the *M_t_*/*M*_∞_ versus √*t* plot, using data from the initial absorption period (1–6 h). The slope *k* of this linear relationship is related to the diffusion coefficient as (5):

(5) $$D={\left(\frac{k\cdot L}{4}\right)}^{2}\cdot \pi$$

where:

*D*—diffusion coefficient, [m^2^ s^−1^],
*k*—slope [s^−1/2^],
*L*—specimen half-thickness, [m].

All calculations were performed assuming one-dimensional diffusion and uniform material properties. The obtained values of the diffusion coefficient represent an approximation valid for the initial diffusion stage and are used for comparative analysis between materials.

The short-time approximation of Fick’s second law is generally considered valid for the initial stage of absorption, typically for *M_t_*/*M*_∞_ ≤ 0.5. Therefore, diffusion coefficients in the present study were estimated using the initial absorption region (1–6 h), where approximately linear behaviour was observed.

The applied Fickian model represents an approximation valid primarily for the initial stage of absorption and was used in the present study for comparative analysis between materials rather than for rigorous determination of absolute diffusion parameters.

## 3. Results

### 3.1. Hardness Results

The measured Shore hardness values of the investigated elastomeric materials ranged from 67.6 ± 0.55 (TPU 60A) to 89.2 ± 0.84 (TPU 98A). A generally increasing trend in measured hardness was observed with increasing nominal TPU grade, indicating generally consistent comparative behaviour between materials. However, minor deviations were observed between certain materials, particularly TPU 82A (84.8 ± 0.45) and TPU 85A (83.6 ± 0.55), where the lower nominal grade showed slightly higher hardness, with a similar trend for TPU 92A (88.2 ± 0.84) and TPU 95A (87.8 ± 0.84). This discrepancy can be attributed to differences in material formulation and manufacturing processes among suppliers. Additionally, a saturation effect was observed at higher hardness levels (≥90A), where differences between materials became less pronounced. The relatively low standard deviation values confirm good repeatability and reliability of the measurements, although increased variability was observed for TPU 89A, suggesting potential material or process-related inconsistencies (Table 3). It should be noted that the specimen thickness used in this study was 2 mm, which is below the minimum thickness specified in ISO 868 for standard Shore hardness testing. Therefore, the measured hardness values should not be interpreted as standardized absolute Shore A values. Instead, they were used primarily for comparative ranking of the investigated FDM-printed specimens measured under identical testing conditions. 

### 3.2. Hygroscopic Behaviour

**Figure 2:**
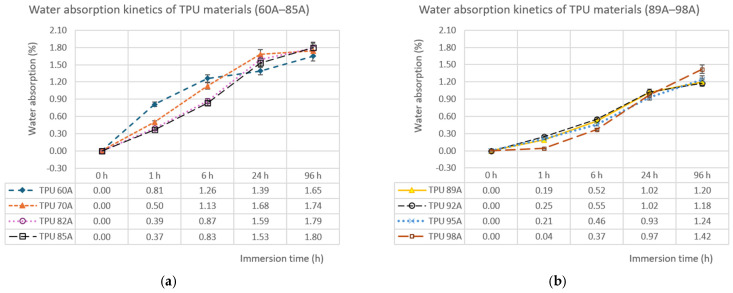
Water absorption kinetics of TPU materials with different Shore A hardness: (**a**) 60A–85A and **(b**) 89A–98A. Error bars represent standard deviation (±SD, *n* = 5). Connecting lines are included as visual guides to illustrate absorption trends and do not represent diffusion model fits.

The water absorption behaviour of the investigated TPU materials revealed a characteristic diffusion-controlled response across the entire hardness range (60A–98A), with a rapid initial uptake followed by a gradual approach to equilibrium (Figure 2a). In the lower hardness group (60A–85A), the absorption kinetics were strongly dependent on material stiffness, with softer materials (60A and 70A) exhibiting faster initial water uptake which may be associated with increased chain mobility and free volume. However, these differences diminished over time, with all materials reaching similar equilibrium absorption values (~1.65–1.80%) after 96 h.

Notably, minor deviations were observed between TPU 82A (84.8 ± 0.45) and TPU 85A (83.6 ± 0.55), where the lower nominal grade exhibited slightly higher measured hardness. This discrepancy indicates that nominal hardness designations do not fully reflect the actual material behaviour and may contribute to differences in hygroscopic response.

In contrast, the higher hardness group (89A–98A) demonstrated more uniform absorption kinetics during the initial immersion period, with only minor differences between materials up to 24 h (Figure 2b). However, pronounced divergence occurred at longer exposure times, where TPU 98A exhibited the highest equilibrium absorption (1.42%), despite its higher nominal hardness. This behaviour suggests that, in higher hardness materials, water absorption may be more strongly influenced by material composition and microstructural characteristics rather than hardness alone.

Overall, the results indicate a transition from behaviour more closely associated with hardness in softer TPU materials to behaviour increasingly influenced by material-specific characteristics in harder TPU grades, highlighting the importance of considering both short-term kinetics and long-term equilibrium behaviour when evaluating flexible polymer materials for engineering and biomedical applications.

**Figure 3:**
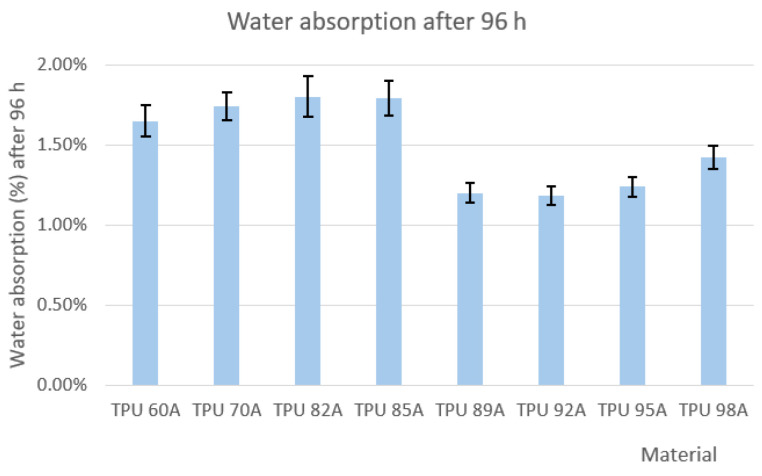
Water absorption after 96 h of 0.9% NaCl solution immersion. Error bars represent standard deviation (±SD, *n* = 5).

The water absorption after 96 h revealed a clear distinction between lower- and higher-hardness TPU materials (Figure 3). Softer materials (60A–85A–82A) exhibited higher absorption values (~1.65–1.80%) after 96 h, while harder materials (89A–95A) showed comparatively lower absorption (~1.18–1.25%). This trend may be associated with increased chain mobility and free volume in softer polymers. However, TPU 98A deviated from this pattern, exhibiting elevated absorption (~1.42%), suggesting that hardness alone is not a sufficient predictor of hygroscopic behaviour.

### 3.3. Dimensional Stability

**Figure 4:**
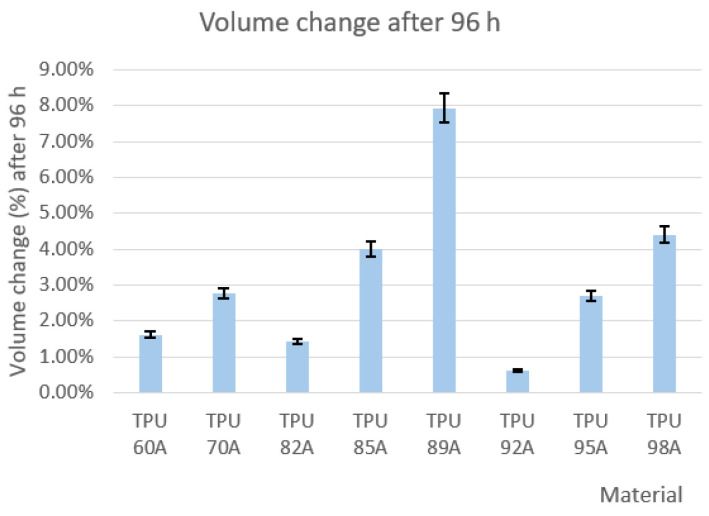
Volumetric expansion of TPU materials after 96 h of 0.9% NaCl solution immersion. Error bars represent standard deviation (±SD, *n* = 5).

The volumetric change after 96 h showed significant variability among the investigated TPU materials and did not directly correlate with water absorption behaviour (Figure 4). The most pronounced expansion was observed for TPU 89A (~8%), despite its moderate water uptake, indicating that swelling behaviour is governed not only by the amount of absorbed water but also by the material’s internal structure. No macroscopic delamination or blistering was visually observed during immersion testing; however, localized internal structural changes cannot be excluded. In contrast, TPU 92A exhibited minimal volumetric change (~0.6%), suggesting higher dimensional stability. Softer materials (60A and 82A) showed relatively low expansion (~1.4–1.6%), while TPU 85A and TPU 98A demonstrated increased swelling (~4–4.5%). These results highlight that the interaction between water and polymer microstructure plays a dominant role in dimensional changes, and that water absorption alone cannot be used as a reliable indicator of swelling behaviour.

### 3.4. Correlation Between Shore Hardness and Water Absorption

**Figure 5:**
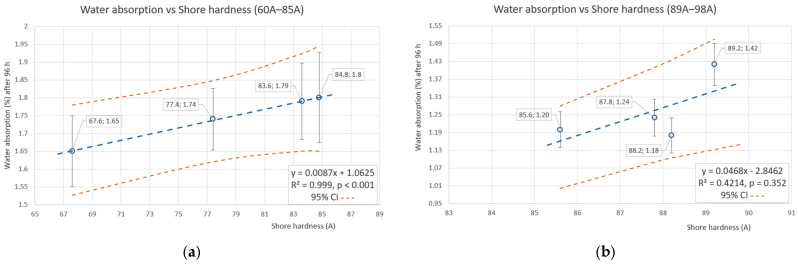
Relationship between Shore hardness and water absorption after 96 h for TPU materials: (**a**) lower hardness range (60A–85A), showing a strong linear correlation (R^2^ = 0.999, *p* < 0.001); (**b**) higher hardness range (89A–98A), showing a weak correlation (R^2^ = 0.4214, *p* = 0.352) and increased data dispersion.

The relationship between Shore hardness and water absorption after 96 h was analysed using regression models for two distinct hardness ranges. For the lower hardness group (60A–85A), a strong linear relationship was observed (R^2^ = 0.999), indicating that water absorption increases proportionally with increasing hardness within this range (Figure 5a). The measured data exhibited a consistent monotonic trend with minimal scatter, indicating a strong association between Shore hardness and hygroscopic behaviour for softer TPU materials.

However, it should be noted that each regression subgroup contained only four materials. Therefore, the obtained R^2^ values should be interpreted primarily as indicative comparative trends rather than as statistically robust predictive relationships. The regression analysis should be interpreted as exploratory due to the limited number of materials within each subgroup.

In contrast, the higher hardness group (89A–98A) demonstrated a markedly weaker correlation (R^2^ = 0.4214), with increased data dispersion and no clear linear dependence between hardness and water absorption (Figure 5b). In particular, TPU 98A exhibited significantly higher absorption compared to other materials within this group, deviating from the general trend. This behaviour indicates that, in higher hardness TPU materials, water absorption does not appear to be primarily associated with hardness alone but rather with material-specific factors such as chemical composition and microstructural characteristics.

Overall, the results reveal a transition in the governing mechanisms of water absorption around 85–90 Shore A, shifting from behaviour closely associated with hardness in softer materials to a regime increasingly associated with material-specific structural characteristics in harder TPU grades. Consequently, a single regression model is not sufficient to describe the entire hardness range, and separate analytical approaches are required for accurate prediction of hygroscopic behaviour.

### 3.5. Diffusion Behaviour

The calculated diffusion coefficients of the investigated TPU materials ranged from 1.40 × 10^−13^ to 3.40 × 10^−12^ m^2^ s^−1^, indicating that all materials exhibit comparable diffusion kinetics within the same order of magnitude (Table 4). The lowest diffusion coefficient was obtained for TPU 98A (1.40 × 10^−13^ m^2^ s^−1^), which extends the overall range by one order of magnitude. The exceptionally low diffusion coefficient obtained for TPU 98A may be influenced by the very low initial absorption values used in the short-time fitting procedure and should therefore be interpreted with caution. No clear monotonic relationship between Shore hardness and diffusion coefficient was observed. The highest diffusion coefficient was obtained for TPU 70A, while the lowest value was recorded for TPU 95A, suggesting that diffusion behaviour may be influenced by material-specific characteristics rather than hardness alone. Furthermore, no clear correlation between the diffusion coefficient and equilibrium water absorption was observed, indicating that diffusion kinetics and equilibrium absorption behaviour may be associated with different mechanisms.

**Figure 6:**
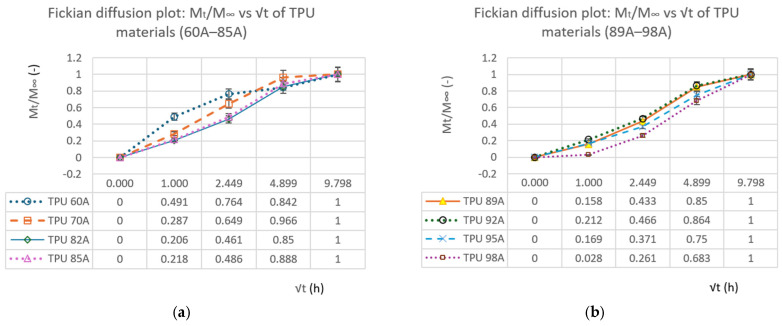
Water diffusion behaviour of flexible TPU materials described by the Fickian diffusion model: (**a**) lower hardness range (60A–85A); (**b**) higher hardness range (89A–98A).

The *M_t_*/*M*_∞_ vs. √*t* plots for TPU materials exhibit an approximately linear behaviour in the initial stage (up to 6 h), suggesting approximately Fickian diffusion behaviour during the early stage of absorption (Figure 6). In the lower-hardness group (60A–85A, Figure 6a), more pronounced differences in the initial slopes are observed, indicating a stronger influence of material stiffness on diffusion kinetics. In contrast, the higher-hardness group (89A–98A, Figure 6b) shows more uniform behaviour during the early stage of absorption, with smaller differences between materials. Notably, TPU 98A exhibits significantly slower initial uptake compared to other materials, followed by convergence at longer times. These results indicate a transition in diffusion behaviour, from hardness-influenced kinetics in softer TPU materials to behaviour increasingly influenced by material-specific structural characteristics in higher-hardness grades.

A complementary power-law analysis (*M_t_*/*M*_∞_ = *kt*^n^) was additionally performed using the initial absorption stage (1–6 h). Most investigated TPU materials exhibited diffusion exponents in the range of *n* ≈ 0.44–0.56, indicating behaviour approximately consistent with Fickian diffusion during the early absorption stage. However, TPU 60A (*n* = 0.247) and particularly TPU 98A (*n* = 1.246) showed noticeable deviations, suggesting that additional material-specific transport mechanisms may contribute to the diffusion behaviour in certain TPU grades.

### 3.6. Optical Microscopy Analysis of Cross-Sectional Pore Morphology

For the microscopic analysis of the samples, an Olympus PME optical microscope (Olympus, Tokyo, Japan) was used at a magnification of 100x to observe the internal microstructure, interlayer bonding, and pore morphology of the FDM-printed specimens. Digital visualization and image acquisition were performed using DinoCapture 2.0 software (Dino-Lite Europe, IDCP B.V., Almere, The Netherlands).

**Figure 7:**
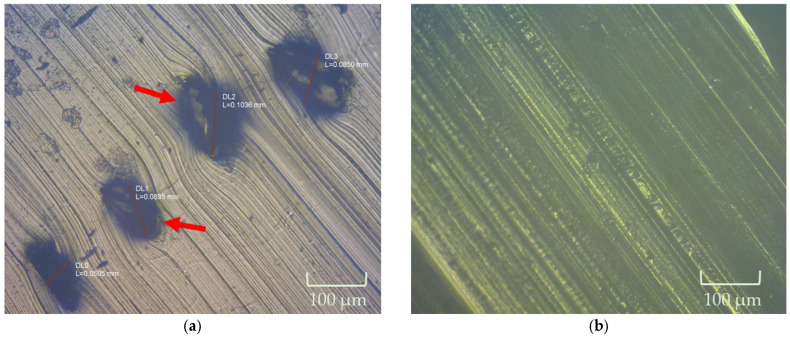
Optical microscopy analysis of interlayer structure and pore morphology in: (**a**) TPU 60A and (**b**) TPU 95A. Representative pore regions are indicated by red arrows.

The microscopy analysis was primarily qualitative (Figure 7). Due to differences in colour, transparency, light reflection, and image contrast among the investigated TPU materials, accurate quantitative evaluation of pore size and pore fraction was not feasible for all samples using optical microscopy alone. However, the obtained micrographs enabled comparative observation of characteristic interlayer structures and general pore distribution trends between the soft TPU group (60A–85A) and the hard TPU group (89A–98A).

Microscopy analysis revealed noticeable differences in the internal morphology between the soft TPU 60A and hard TPU 95A materials. In TPU 60A, distinct dark pore-like regions were observed between the deposited FDM layers, indicating increased internal porosity and a less compact interlayer structure. Representative pore-like defects observed in TPU 60A exhibited maximum characteristic dimensions ranging from approximately 50 to 104 µm under the applied optical magnification conditions. Approximate image-based pore area analysis indicated a pore fraction of approximately 8.6% for TPU 60A. These pores may facilitate moisture penetration and diffusion through preferential transport pathways within the material. In contrast, TPU 95A exhibited a more uniform and compact layered morphology with no clearly visible pore defects under the applied optical magnification conditions, suggesting improved interlayer bonding and reduced internal void formation. The observed structural differences are consistent with the lower water absorption behaviour observed in harder TPU materials.

## 4. Discussion

The results of this study indicate that the hygroscopic behaviour of FDM-printed TPU materials is influenced by multiple interacting factors, including material hardness, composition, and internal structure. Such a multi-factorial response is consistent with the broader understanding that both material-related and process-related parameters influence the performance of additively manufactured polymers [1,2,7,16].

In TPU systems, moisture transport and dimensional stability may be affected by factors such as soft-segment mobility, hard-segment hydrogen bonding, and microphase morphology. Variations in polyol chemistry and hard/soft segment distribution may therefore contribute to differences in water absorption and swelling behaviour between materials, particularly in cases where volumetric expansion does not directly correlate with equilibrium water uptake.

For the lower hardness group (60A–85A), a strong linear relationship between Shore hardness and water absorption was observed, indicating that hardness is associated with hygroscopic behaviour within this range. This trend is consistent with the interpretation that differences in polymer chain mobility and free volume contribute to water transport in flexible polymer systems [2,7,17].

In contrast, the higher hardness group (89A–98A) exhibited a weak and non-linear relationship between hardness and water absorption. The increased scatter of results, together with the deviation observed for TPU 98A, indicates that hardness alone is insufficient to describe material behaviour in this regime. These findings indicate that water absorption in this hardness range is more closely associated with material-specific characteristics such as chemical composition and microstructural features, including phase morphology and the distribution of hard and soft segments within the TPU structure [2,5,7,17].

Optical microscopy revealed visible interlayer pores in TPU 60A, with characteristic dimensions in the range of approximately 50–100 µm. Approximate image-based pore area analysis indicated a pore fraction of approximately 8.6% for TPU 60A.

Such pore sizes are consistent with interlayer void dimensions commonly reported for FDM/FFF fabricated polymer structures. In contrast, TPU 95A exhibited a more compact and homogeneous layered morphology without clearly visible pore defects under the applied optical magnification conditions [18].

The present microscopy analysis was primarily qualitative in nature. More advanced characterization methods, such as high-resolution SEM imaging or micro-CT analysis, were beyond the scope of the present study and should be considered in future investigations to enable quantitative evaluation of porosity, interlayer morphology, and internal structural heterogeneity.

The observed interlayer pore morphology suggests the presence of preferential diffusion pathways that may contribute to moisture transport behaviour in softer TPU materials.

The statistical interpretation of the regression analysis should also be considered with caution. Although the lower-hardness group exhibited an apparently strong linear relationship (R^2^ = 0.999), each subgroup consisted of only four commercially distinct materials. Consequently, the regression results should be interpreted as exploratory indicators of possible behavioural trends rather than statistically definitive evidence of underlying mechanisms. The separation into lower- and higher-hardness material groups was introduced primarily as a comparative interpretative framework based on the observed experimental trends and should not be interpreted as a statistically validated change-point model. Additional studies including larger material populations and controlled material formulations would be required to establish more rigorous structure–property relationships.

A key finding of this study is the absence of a clear correlation between water absorption and volumetric change. For example, TPU 89A exhibited the highest volumetric expansion despite only moderate water uptake, whereas TPU 92A showed minimal dimensional change. This suggests that swelling behaviour is not solely dependent on the amount of absorbed water but is also influenced by the interaction between water molecules and the polymer network. Similar behaviour has been reported in studies on moisture-related ageing and dimensional instability of polymer systems, where absorption capacity and structural response are not directly coupled [8,19,20].

The volumetric calculations were based on simplified geometric relations assuming approximately uniform dimensional changes. However, due to the layered architecture of FDM-printed materials, anisotropic swelling behaviour may occur and could influence the calculated volumetric expansion values.

It should be noted that all experiments were conducted at room temperature (22 ± 1 °C), whereas physiological conditions correspond to approximately 37 °C. Since temperature is known to influence diffusion kinetics, the obtained results may underestimate diffusion rates under in vivo conditions, and this aspect should be considered when interpreting the applicability of the results for biomedical use.

Furthermore, the present study focused exclusively on hygroscopic behaviour and diffusion characteristics and did not include biological evaluation such as cytotoxicity, biocompatibility, additive leaching, or sterilization stability. Therefore, the biomedical relevance of the investigated materials should be interpreted only in the context of simplified moisture-exposed environments rather than as direct validation for biomedical use.

Additionally, the diffusion analysis indicates that transport kinetics and equilibrium behaviour appear to be influenced by different mechanisms. Although the calculated diffusion coefficients for most materials were of the similar order of magnitude (10^−12^ m^2^ s^−1^), no clear monotonic relationship with hardness was observed. In addition, only a weak correlation between diffusion coefficient and equilibrium absorption was identified, suggesting that diffusion rate and sorption capacity are not directly coupled. This distinction between transport kinetics and overall uptake is consistent with previous studies on moisture transport in polymeric and additively manufactured systems [8,19,20].

In the present study, the equilibrium absorption value *M*_∞_ was approximated using the 96 h absorption value. Since a complete saturation plateau was not fully established for all materials, the calculated diffusion coefficients should be interpreted as comparative estimates rather than absolute material constants.

The diffusion analysis in this study is based on a one-dimensional Fickian model using the specimen half-thickness as the characteristic diffusion length. This assumption is justified by the geometry of the specimens, which are thin sheets (2 mm thickness), where moisture transport is expected to be dominated by the through-thickness direction.

However, due to the layered structure inherent to FDM manufacturing, anisotropic transport behavior may occur, particularly along interlayer interfaces that can act as preferential diffusion pathways. This effect was not directly quantified in the present study and therefore represents a limitation.

Future work should include a systematic investigation of diffusion as a function of print orientation and layer alignment to better capture anisotropic effects and establish a more complete understanding of moisture transport in additively manufactured polymer systems.

A complementary power-law analysis performed in the present study supported approximately Fickian behaviour during the early absorption stage for most investigated materials. Nevertheless, additional non-Fickian and extended transport modelling approaches may provide further insight into moisture transport mechanisms in flexible TPU materials.

Processing conditions may also contribute to the observed variability between materials. Differences in extrusion temperature and printing parameters can influence interlayer bonding, porosity, and microstructural homogeneity, which in turn affect water diffusion behaviour. In particular, higher extrusion temperatures may improve interlayer adhesion and reduce internal void content, potentially limiting fluid penetration into the material. Although all specimens were produced under comparable conditions following manufacturers’ recommendations, slight variations in optimal processing parameters between materials may have influenced the results. These effects should therefore be interpreted as potential contributing factors rather than directly validated causes. Previous studies have similarly shown that print-related parameters such as infill density, surface texture, orientation, and process-induced internal structure significantly affect the behaviour of additively manufactured polymer components [1,2,10,11,12,16].

A limitation of the present study is that the investigated TPU materials were sourced from multiple manufacturers and therefore differ not only in Shore hardness, but potentially also in chemical composition, hard/soft segment ratio, polyol chemistry, additives, and thermal history. Since direct chemical and thermal characterization (e.g., FTIR, DSC, density analysis) was not performed, the observed relationships between hardness and hygroscopic behaviour should be interpreted as comparative phenomenological trends rather than definitive causal relationships. Future studies should therefore investigate chemically matched TPU series or include complementary structural characterization to isolate the specific contribution of hardness.

The obtained equilibrium water absorption values (~1.18–1.80%) fall within the range reported for FDM-processed TPU and related flexible polymer systems, where moisture uptake typically depends on both material composition and processing conditions [5,7]. Similarly, the calculated diffusion coefficients obtained in the present study (10^−13^–10^−12^ m^2^ s^−1^) are comparable to values reported for TPU and flexible polyurethane systems in previous studies. Reported diffusion coefficients for water transport in TPU materials and related elastomeric polymer systems typically fall within the range of approximately 10^−13^ to 10^−12^ m^2^ s^−1^ depending on specimen geometry, temperature, crystallinity, and processing-induced morphology [8,19,20]. Comparable diffusion behaviour has also been reported for FDM-processed polymer structures, where interlayer porosity and print-induced anisotropy may influence moisture transport kinetics. These observations support the applicability of the Fickian diffusion model to the initial stage of water uptake and confirm that the observed transport behaviour is physically consistent with established diffusion mechanisms in polymer materials.

The investigated materials are relevant for biomedical applications such as patient-specific anatomical models, dental surgical guides, and soft-tissue analogues used in preoperative planning and surgical training. In these applications, dimensional stability and moisture interaction are important factors influencing performance and accuracy.

In the present study, hygroscopic behaviour was evaluated using static immersion in a 0.9% NaCl solution, which represents a simplified physiological environment. This approach provides a controlled and reproducible framework for isolating diffusion-driven moisture uptake and comparing material behaviour.

However, real physiological conditions are significantly more complex and involve additional factors such as proteins, pH variations, enzymatic activity, and dynamic mechanical loading. These factors may influence both diffusion kinetics and equilibrium absorption through mechanisms such as protein adsorption, swelling, and mechanically induced transport.

Therefore, the adopted methodology can be considered a simplified and partially conservative model, but it does not fully capture in vivo conditions. Future research should focus on more physiologically relevant environments, including protein-containing solutions, controlled pH conditions, and cyclic loading, to better approximate real biomedical applications.

Overall, the present results extend previous studies by demonstrating that the relationship between hardness and hygroscopic behaviour is not uniform across the entire material range. Instead, a transition is observed from behaviour that appears more closely associated with hardness in softer TPU materials to behaviour that appears to be increasingly influenced by material-specific structural characteristics in higher-hardness grades.

In contrast to previous studies that typically consider water absorption or diffusion behaviour independently, this work provides a combined analysis of diffusion kinetics, equilibrium absorption, and dimensional stability, demonstrating that these properties are not directly coupled. In this way, the study contributes to a broader comparative understanding of structure–property relationships in flexible FDM-printed polymers by integrating material-related, process-related, and transport-related effects into a unified framework.

## 5. Conclusions

This study systematically investigated the hygroscopic behaviour of TPU materials fabricated using FDM across a wide range of Shore hardness values.

The study indicates that the relationship between Shore hardness and hygroscopic behaviour in FDM-printed TPU materials exhibits two distinct regimes. For softer TPUs (60A–85A), a strong linear correlation was observed (R^2^ = 0.999), indicating that Shore hardness may serve as an indicative predictor of moisture absorption within a limited hardness range.

In contrast, for harder TPUs (89A–98A), the relationship becomes weak and non-linear, suggesting that hardness alone is insufficient and that microstructural factors such as porosity and interlayer bonding may contribute significantly to the observed behaviour. From a practical perspective, Shore hardness may therefore serve as a useful design parameter for lower-hardness TPU materials (60A–85A), where a strong linear relationship between hardness and moisture absorption was observed. In contrast, for higher-hardness grades (89A–98A), where the relationship became weak and non-linear, additional microstructural characterization and experimental validation are required.

The diffusion analysis revealed that most materials exhibited diffusion coefficients within the order of 10^−12^ m^2^ s^−1^, while TPU 98A showed a lower value in the order of 10^−13^ m^2^ s^−1^, with no clear monotonic dependence on hardness. The lowest diffusion coefficient was obtained for TPU 98A (1.40 × 10^−13^ m^2^ s^−1^), which extends the overall range by one order of magnitude. Additionally, no direct coupling was observed between diffusion kinetics, equilibrium water absorption, and volumetric change, indicating that these properties may be associated with different mechanisms.

The results further suggest that process-induced structural characteristics may contribute to moisture transport behaviour in FDM-printed materials.

The observed differences in interlayer pore morphology additionally suggest that internal structural characteristics may play an important role in governing moisture transport in FDM-printed TPU materials.

Overall, the findings demonstrate that hygroscopic behaviour in flexible TPU materials cannot be reliably predicted based solely on Shore hardness. Instead, a transition from hardness-dependent behaviour to structure-influenced behaviour is observed, emphasizing the need for a multi-parameter approach when evaluating material performance.

These findings provide comparative insight into the structure–property relationships associated with moisture interaction in flexible polymers under simplified moisture-exposed conditions relevant to engineering and biomedical-related environments.

Future research should focus on direct microstructural characterization and its relationship with diffusion behaviour, including SEM, micro-CT, DSC, FTIR, and porosity analysis. Future studies should also investigate chemically comparable TPU materials from a single manufacturer and include testing under physiologically relevant conditions to better evaluate long-term hygroscopic and biomedical performance.

## Figures and Tables

**Table 1:** Flexible polymer filaments selected for the experimental investigation.

Manufacturer	Recreus (Alicante, Spain)	Recreus (Alicante, Spain)	Recreus (Alicante, Spain)	AzureFilm (Sežana, Slovenia)	Plastika Trček (Ljubljana, Slovenia)	Fillamentum (Hulín, Czech Republic)	Elegoo (Shenzehn, China)	AzureFilm (Sežana, Slovenia)
Commercial Name	FilaFlex 60A	FilaFlex 70A	FilaFlex 82A	TPU Flexible 85A	TPU Flexible 89A	Flexfill TPU 92A	TPU 95A	TPU Flexible 98A
Hardness (Shore A)	60	70	82	85	89	92	95	98

**Table 2:** Printing parameters.

Material	Layer Height	Infill	Infill Pattern	Print Speed	Nozzle Temp (1st Layer)	Nozzle Temp (Other Layers)	Bed Temperature
Filaflex TPU 60A (Recreus, Alicante, Spain)	0.2 mm	100%	rectilinear	40 mm/s	225 °C	225 °C	50 °C
Filaflex TPU 70A (Recreus, Alicante, Spain)	0.2 mm	100%	rectilinear	40 mm/s	235 °C	230 °C	50 °C
Filaflex TPU 82A (Recreus, Alicante, Spain)	0.2 mm	100%	rectilinear	40 mm/s	250 °C	240 °C	50 °C
Azurefilm TPU 85A (AzureFilm, Sežana, Slovenia)	0.2 mm	100%	rectilinear	30 mm/s	240 °C	240 °C	50 °C
Plastika Trček TPU 89A (Plastika Trček, Ljubljana, Slovenia)	0.2 mm	100%	rectilinear	30 mm/s	245 °C	245 °C	50 °C
Flexfill 92A TPU (Fillamentum, Hulín, Czech Republic)	0.2 mm	100%	rectilinear	45 mm/s	245 °C	248 °C	50 °C
Elegoo 95A TPU (Elegoo, Shenzehn, China)	0.2 mm	100%	rectilinear	45 mm/s	245 °C	248 °C	50 °C
Azurefilm 98A TDS (AzureFilm, Sežana, Slovenia	0.2 mm	100%	rectilinear	30 mm/s	240 °C	242 °C	50 °C

**Table 3:** Shore hardness (A) of tested elastomeric materials (mean ± SD, *n* = 25).

Material	TPU60A	TPU70A	TPU82A	TPU85A	TPU89A	TPU92A	TPU95A	TPU98A
Mean ($\bar{x}$)	67.6	77.4	84.8	83.6	85.6	88.2	87.8	89.2
±SD	0.55	0.55	0.45	0.55	1.90	0.84	0.84	0.84

**Table 4:** Water absorption at 96 h and corresponding diffusion coefficients.

Material	TPU 60A	TPU 70A	TPU 82A	TPU 85A	TPU 89A	TPU 92A	TPU 95A	TPU 98A
Absorption	1.65	1.74	1.80	1.79	1.20	1.18	1.24	1.42
*D*, (m^2^ s^−1^)	1.93 × 10^−12^	3.40 × 10^−12^	1.70 × 10^−12^	1.87 × 10^−12^	1.96 × 10^−12^	1.68 × 10^−12^	1.06 × 10^−12^	1.40 × 10^−13^

Absorption—Water absorption at 96 h, *M*_∞_ (%), *D*—Diffusion coefficient, *D* (m^2^ s^−1^).

## Data Availability

The data presented in this study are publicly available at Zenodo: https://doi.org/10.5281/zenodo.20409839.

## Abbreviations

The following abbreviations are used in this manuscript:

TPU	Thermoplastic polyurethane
FDM	Fused deposition modeling
AM	Additive manufacturing
SEBS	Styrene-ethylene-butylene-styrene
TPE	Thermoplastic elastomer
